# Suitable Cathode NMP Replacement for Efficient Sustainable Printed Li-Ion Batteries

**DOI:** 10.1021/acsaem.1c02923

**Published:** 2022-03-29

**Authors:** Rafal Sliz, Juho Valikangas, Hellen Silva Santos, Pauliina Vilmi, Lassi Rieppo, Tao Hu, Ulla Lassi, Tapio Fabritius

**Affiliations:** † Optoelectronics and Measurement Techniques Unit, University of Oulu, 90570 Oulu, Finland; ‡ Research Unit of Sustainable Chemistry, University of Oulu, 90570 Oulu, Finland; § Fibre and Particle Engineering Research Unit, University of Oulu, 90570 Oulu, Finland; ∥ Research Unit of Medical Imaging, Physics and Technology, University of Oulu, 90570 Oulu, Finland

**Keywords:** printed batteries, NMP, DMF, solvent, NMC, NMC88, NMC523, screen printing

## Abstract

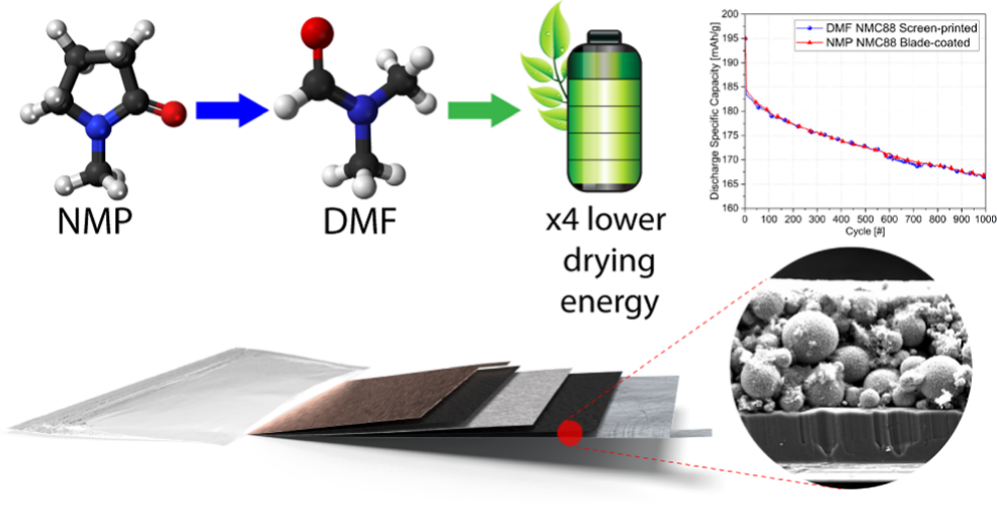

*N*-methyl-2-pyrrolidone (NMP) is the most common solvent for manufacturing cathode electrodes in the battery industry; however, it is becoming restricted in several countries due to its negative environmental impact. Taking into account that ∼99% of the solvent used during electrode fabrication is recovered, dimethylformamide (DMF) is a considerable candidate to replace NMP. The lower boiling point and higher ignition temperature of DMF lead to a significant reduction in the energy consumption needed for drying the electrodes and improve the safety of the production process. Additionally, the lower surface tension and viscosity of DMF enable improved current collector wetting and higher concentrations of the solid material in the cathode slurry. To verify the suitability of DMF as a replacement for NMP, we utilized screen printing, a fabrication method that provides roll-to-roll compatibility while allowing controlled deposition and creation of sophisticated patterns. The battery systems utilized NMC (LiNi*_x_*Mn*_y_*Co*_z_*O_2_) chemistry in two configurations: NMC523 and NMC88. The first, well-established NCM523, was used as a reference, while NMC88 was used to demonstrate the potential of the proposed method with high-capacity materials. The cathodes were used to create coin and pouch cell batteries that were cycled 1000 times. The achieved results indicate that DMF can successfully replace NMP in the NMC cathode fabrication process without compromising battery performance. Specifically, both the NMP blade-coated and DMF screen-printed batteries retained 87 and 90% of their capacity after 1000 (1C/1C) cycles for NMC523 and NMC88, respectively. The modeling results of the drying process indicate that utilizing a low-boiling-point solvent (DMF) instead of NMP can reduce the drying energy consumption fourfold, resulting in a more environmentally friendly battery production process.

## Introduction

In recent years, batteries have become a crucial element of our everyday life. Their pervasiveness is caused by a continuously increasing amount of portable devices with higher computing power, larger displays, and improved communication capabilities.^1^ Within the scope of the internet of things (IoT) and 4th Industrial Revolution, manufacturers are continuously introducing immense amounts of battery-operated electronic devices in the market.^2^ From another perspective, increasing environmental awareness and ambitious CO_2_ emission reduction goals across the world stimulate a transition from combustion engine-powered vehicles toward battery-powered electric solutions.^3,4^ Additionally, the concept of a sustainable smart grid, where the energy generation and storage systems are distributed, requires augmented battery systems capable of storing energy for periods of increased energy generation and provide additional capacity during elevated power consumption.^5^ Regardless of application, current technological advancements strongly rely on battery systems that are expected to be inexpensive, lightweight, small, highly energy-dense, capable of rapid charge–discharge, long-lasting, and safe.^6^

**Figure 1:**
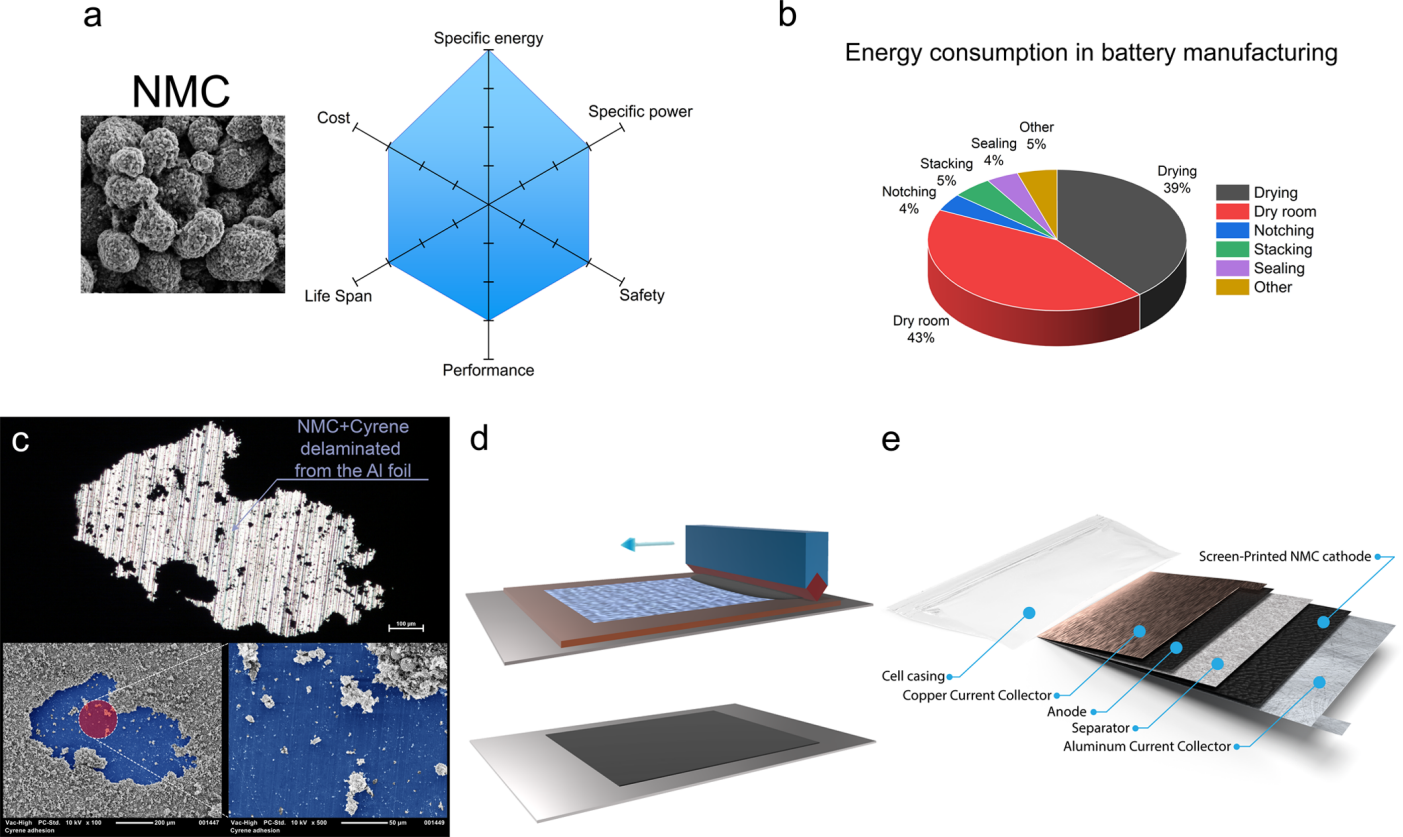
NMC screen-printed batteries. (a) Characteristics of NMC chemistry. (b) Energy consumption occurring during battery manufacturing with cathodes based on the NMP solvent.^10^ Consumption estimates for drying include both energy needed to evaporate and recover the NMP solvent. (c) Optical microscopy and SEM insets demonstrating delamination of printed cyrene-based NMC materials from the surface of an aluminum current collector (in the insets, Al is colored blue). (d) Sketch demonstrating screen printing of the cathode on the aluminum current collector. (e) Fabricated pouch cells that underwent cycling and characterization.

The NMC cathode chemistry (lithium-nickel-manganese-cobalt-oxide (LiNi*_x_*Mn*_y_*Co*_z_*O_2_)) of lithium-ion (Li-ion) batteries belongs to one of the most successful battery systems obtained by combining nickel, manganese, and cobalt at various ratios. Batteries with NMC-based cathode chemistry demonstrate excellent overall performance: high specific energy, low self-heating, and necessary lifespan (Figure 1a). However, in addition to the economic and performance-related factors, the sustainability of batteries becomes a critical aspect that needs to be taken into account.^7^

Data collected by Davidsson provides evidence suggesting that a 100 kWh battery utilized in a long-range vehicle requires 5–6.5 MWh of energy for fabrication in a large-scale, well-optimized process (without mining and raw material processing).^8^ In addition, production of a cathode for a 100 kWh battery involves ∼100 L of the *N*-methyl-2-pyrrolidone (NMP) solvent that is used in the cathode slurry formulation.^9^ Although ∼99% of the NMP is recovered and reused in the battery fabrication process, its relatively high boiling point (202 °C) and low autoignition temperature (252 °C) impose increased energy demands and safety concerns during the electrode drying process. Researchers point out that the NMP drying-recovery processes require ∼40% of the energy needed for battery manufacturing (Figure 1b).^10−13^

NMP is commonly used as a solvent in pharmaceutical production, chemical processing, and electronics, especially in the battery industry, due to its excellent chemical and thermal stability.^14−16^ In batteries, this dipolar aprotic solvent is used to dissolve polyvinylidene fluoride (PVDF)—the most frequently utilized binder in the cathode slurry formulation.^17^ The low surface tension of NMP (41 mN/m) allows enhanced wetting of the cathode material slurries onto the current collector, consequently improving adhesion.^18,19^

Despite excellent cathode slurry suitability (solvability and low volatility), the toxicity of NMP has come under increasing environmental scrutiny by regulatory bodies in various countries. The United States Environmental Protection Agency labeled NMP as a developmental toxicant. In Europe, due to its classification as a reproductive toxin, NMP is regarded as a substance of very high concern. In 2018, NMP was added to the Registration, Evaluation and Authorization of Chemicals (REACH) list, restricting its consumer application usage to <0.3%.^20^

There have been numerous studies to investigate the potential replacement of NMP.^21^ Aqueous processing of battery electrodes is one of the most commonly applied approaches.^18,22,23^ In addition to resolving the problem of NMP toxicity, water significantly improves the energy efficiency and safety of the fabrication process, thanks to its low boiling point (100 °C). Nonetheless, water processing of cathodes introduces two major challenges. The first concern is related to the need of replacing the PVDF binder with water-soluble alternatives that provide similar performance of the working electrodes, such as high thermal stability, excellent adhesion, flexibility, and R2R process compatibility.^18,24^ The second concern is related to undesired interactions of water with the active material of electrodes. The cathode surface exposed to water/moisture is prone to delithiation of the oxide and formation of lithium salts.^25^ Skrob et al. propose another explanation to electrode deterioration, namely, the proton-lithium exchange.^26^ The degradation due to aqueous processing is especially noticeable for high-Ni-ratio materials.^27,28^ From the processing viewpoint, the high surface tension of water introduces another set of difficulties related to wettability of the slurries and cracking of NMC layers.^29^

In addition to water, a number of alternative approaches have been developed. Some of them include replacement of NMP with DMSO, cyrene, or γ-valerolactone.^30−33^ Another method involves solvent-free fabrication, where the cathode material is sprayed-on or hot-pressed to the aluminum current collector.^34^

Although the proposed “greener” solutions provide encouraging results, they introduce challenges and shortcomings that significantly limit their implementation. For instance, usage of DMSO is linked to the insertion of sulfur impurities that negatively affect the performance of the NMC cathodes.^31^ From the other perspective, green solvents such as cyrene and γ-valerolactone provide promising solvability but the manufactured cathode layers suffer from poor adhesion that affects the battery performance in terms of specific capacity during cycling or Coulombic efficiency, to name a few.^32,33^Figure 1c shows poor adhesion and NMC material delamination from the aluminum current collector surface for the screen-printed cyrene-based NMC slurries. Lastly, the current greener solvents have higher boiling point temperatures than NMP, increasing the energy consumption related to the drying process and solvent recovery (Table 1).

**Table 1:** Comparison of Properties of Solvents Used for the NMC Cathode Slurry

	boiling point [°C]	autoignition temperature [°C]	surface tension [mN/m at 25 °C]	viscosity [mPas]
NMP	202	252	41	1.66
DMSO	189	300	44	1.99
γ-valerolactone	207	524	29.4	2.18
cyrene	226	296	72.5	14.5
DMF	155	445	37.1	0.92

This work focuses on the usage of dimethylformamide (DMF) as a suitable solvent for NMC cathode fabrication. DMF is widely applied in industries related to agricultural chemicals, oil and gas, fibers and textiles, polymers, and refining.^35^ DMF belongs to a group of polar aprotic solvents of similar properties as NMP; the polarity (6.4) and dielectric constant (36.7) of DMF are similar to those of NMP (6.7 and 32.2, respectively), allowing similar slurry/ink formulation. Also, DMF dissolves the PVDF equally well as NMP.^36^ However, from the cathode fabrication point of view, DMF offers four significant advantages: lower boiling point that reduces the energy consumption during cathode drying, very high autoignition temperature that decreases the safety concerns, low surface tension that improves wettability and the adhesion of cathode materials to the current collector, and low viscosity that enables formulation of more NMC-concentrated slurries (Table 1). Although DMF is also considered a toxic solvent and has been recently added to the candidate list of substances of very high concern, two important aspects need to be taken into account: a closed-circuit cathode drying process that allows 99% solvent recovery and relatively low toxicity of DMF-based deposition methods compared to other semiconductor technologies, for example, the fabrication of solar cells.^37,38^

In addition to replacing NMP with DMF, this work utilizes printed electronics as a suitable and sustainable method of fabricating batteries.^39^ In addition to selectivity, flexibility, and electronics hybridization, printing is a unique method that enables fabrication of batteries, generating a negligible amount of material waste.^40^ Thanks to upscaling potential and roll-to-roll (R2R) compatibility, printing can provide large fabrication capacity to produce battery elements and systems at nominal costs.^41^

To the best of the authors’ knowledge, the experimental work presented here provides one of the first investigations into screen printing of NMC523 and NMC88 cathode materials by utilizing DMF as a slurry solvent (Figure 1c). To prove the suitability of DMF, we utilized NMC523 (LiNi_0.5_Mn_0.2_Co_0.3_O_2_) as a well-established reference material. At the same time, a novel NMC88 (LiNi_0.88_Mn_0.03_Co_0.09_O_2_) high-capacity material, with a significantly higher Li/Ni ratio, was used to further demonstrate the potential of the proposed method. For both cathode materials, we analyzed the DMF screen-printed and NMP blade-coated cathodes and pouch batteries. The physicochemical analysis of the printed and blade-coated NMC films was followed by the assembly of coin and pouch cells that underwent 1000 charge–discharge cycles (Figure 1d). In addition, we numerically compared the drying time and related energy savings for NMP and DMF solvents. This contribution offers a solution to reduce the energy consumption during battery fabrication and usage of NMP through the replacement with DMF, enabling broad applicability and immediate implementation, thanks to the screen-printing compatibility.

## Experimental Section

### Printing and Slurry Preparation

The printing trials were conducted using an Ekra E2 printer with Koenen stencil (VA 165–0.05 mm, W-Ø × 22.5°). First, the PVDF (Kureha #1100) was predissolved with the respective solvent (NMP (Alfa Aesar, anhydrous, 99.5%) and DMF (Merck, anhydrous, 99.8%)) and magnetically stirred for 6 h at 40 °C. Consequently, the carbon black (Timcal C45) and the active material were added and mixed in a planetary centrifugal bubble-free mixer (Thinky ARE 250) for 20 min at 2000 rpm. The active material (NMC523 and NMC88) was mixed with PVDF binders and conductive carbon black in a weight ratio of (92:4:4) and concentration of ∼1.27 g/mL. After mixing, the liquid-like slurry was similar to the slurries reported by Wang et al.^42^ Accordingly, the NMP-based slurry was used to blade-coat the aluminum current collector, with a foil thickness of 25 μm, and dried at 80 °C for 1 h. The DMF-based slurry was printed with the Ekra printer at a speed of 30 mm/s and dried at 80 °C for 1 h. Moreover, to test the screen-printer compatibility, a slurry with the cyrene solvent (same protocol as for DMF) was prepared and screen-printed on the surface of the aluminum current collector. To remove any solvent residues, all cathodes were further dried in vacuum at 120 °C for 12 h. After drying, the fabricated films were used for characterization and battery assembly. Samples designated for battery assembly were calendered three times (MTI Hot Rolls Press HR-02). Visual inspection of the samples immediately revealed that the samples using cyrene as the solvent demonstrate poor adhesion (Figure S9) and therefore were not used for assembling batteries. In addition, to investigate the solvability of PVDF in NMP and DMF, two sample solutions (PVDF concentration 4% wt.) with NMP and DMF were magnetically stirred for 6 h at 40 °C, casted at the surface of the aluminum foil, and dried at 50 °C for 1 h. The PVDF films were consequently analyzed using SEM.

### Cathode Characterization

**Figure 2:**
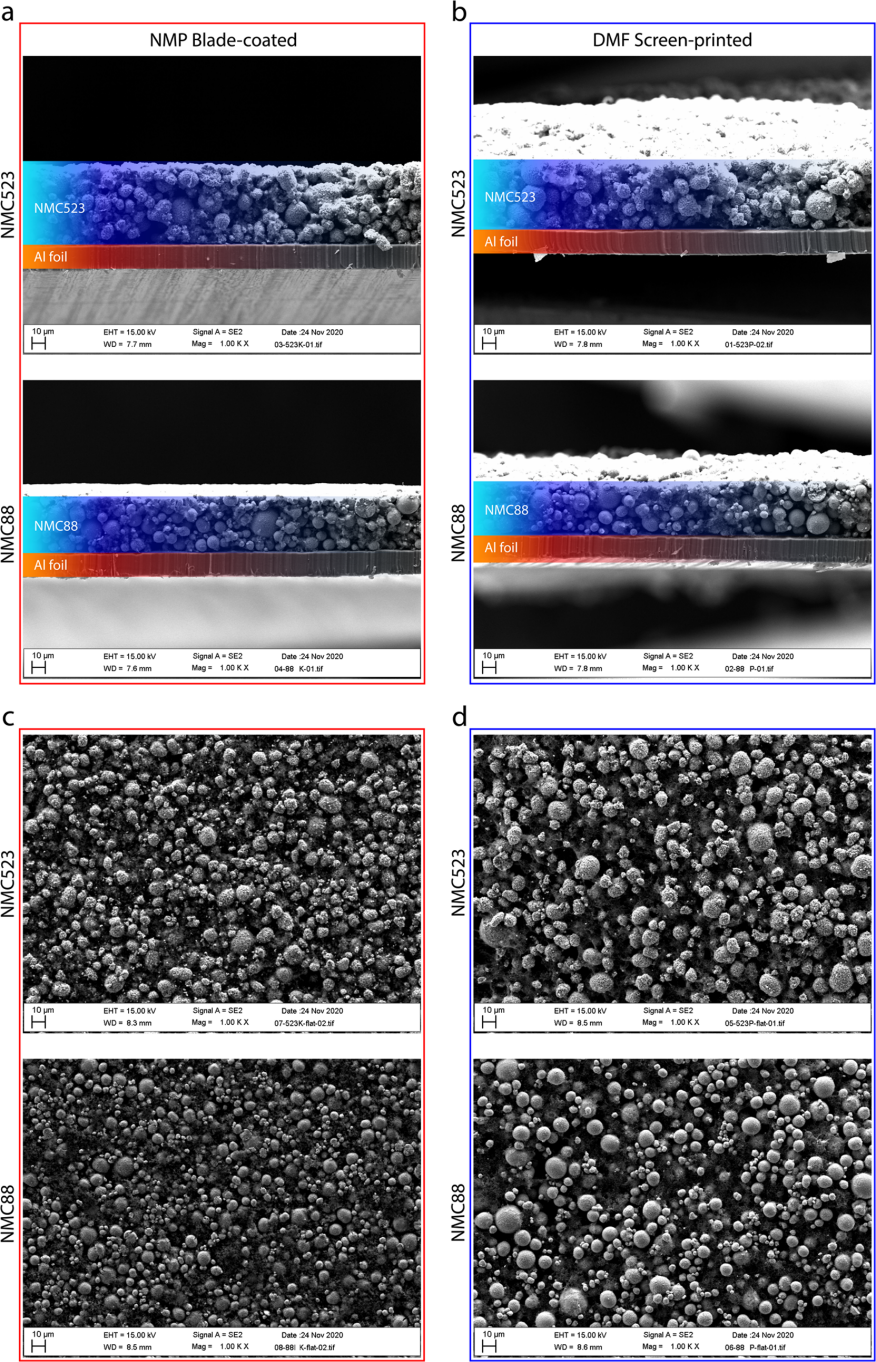
SEM characterization of screen-printed and blade-coated samples before calendering: (a) cross-sectional view of the NMP blade-coated NMC layers; (b) cross-sectional view of the DMF screen-printed NMC layers; (c) plane-view of the NMP blade-coated NMC layers; and (d) plane-view of the DMF screen-printed NMC layers.

**Figure 3:**
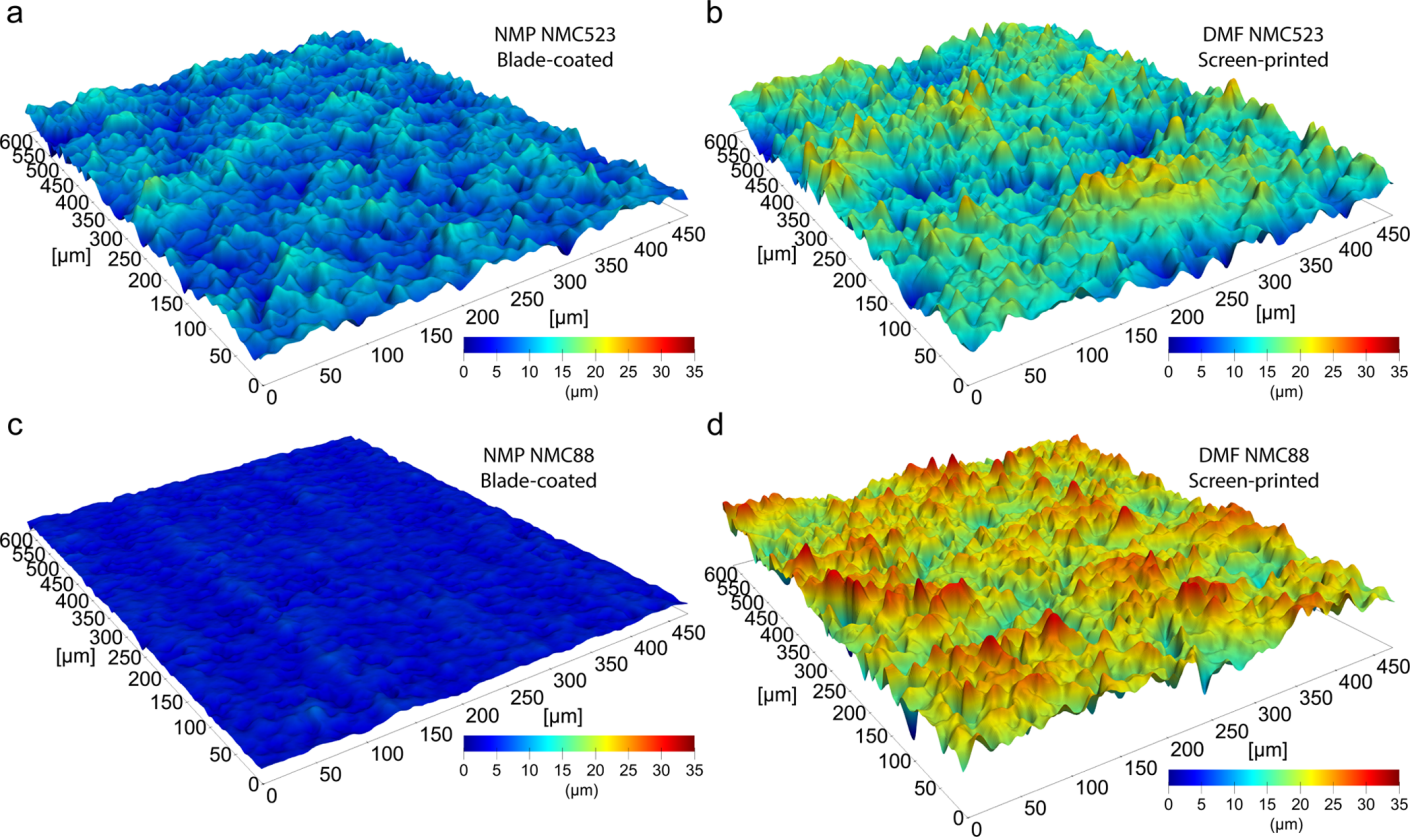
Surface morphology characterization of NMC layers that consisted of different solvents and deposition methods: (a) morphology of NMP NMC523 blade-coated layer; (b) morphology of DMF NMC523 screen-printed layer; (c) morphology of NMP NMC88 blade-coated layer; and (d) morphology of DMF NMC88 screen-printed layer.

The scanning electron microscopy (SEM) surface and cross-sectional images (Figures 2, S1–S3, and S9) of the fabricated NMC films were performed using the electron microscope Zeiss ULTRA plus FESEM. The morphology of the surface of the fabricated NMC layers depicted in Figure 3, S1, and S2 was obtained using a Bruker ConturGT optical profilometer in VSI mode. The images were processed in the Gwyddion software and the final figures rendered with the Paraview software. The crystalline structures of the phases present in NMC materials were studied with X-ray powder diffraction (XPD) measurements at room temperature, performed with a Pananalytical instrument at 45 kV and 40 mA (model X’pert 3 MRD), using an image plate detector and Cu Kα radiation (Kα1 = 1.54 Å). The measurements were conducted at a scan rate of 0.0167°/min in the range of 10–80° (2θ) and 0.017° 2Θ/step. Phase identification and Rietveld refinement were conducted with PDXL V.2 software (Rigaku, Japan) and a PDF-4+ 2020 database. The FTIR characterization was conducted with a ThermoFisher Nicolet iS5 FTIR spectrophotometer in the range of 4000–400 cm^–1^ in ATR mode with a diamond crystal. The TGA characterization was conducted with TA Instruments SDT 650 device (alumina crucible) in the temperature range of 30–1000 °C with a ramp of 10 °C/min, sample volume of ca. 100 μl, and N_2_ flow rate of 100 ml/min. The electron probe microanalysis was performed using JEOL JXA-8530FPlus device. The acquired WDS maps were further transposed into the positions in an equilateral triangle by OriginPro software. To calculate the cathode parameters (porosity, active material loading, and cathode–anode balancing), the thickness and weight of the calendered cathodes were measured with an electronic micrometric screw (Schut, precision ±1 μm) and laboratory high-precision balance (Ohaus Voyager Pro VP214C). The results are provided in Table S2. The pouch cells were balanced using specific capacities of NMC523—180 mAh/g, NMC88—230 mAh/g, and graphite—372 mAh/g. Cells were balanced so that the anode capacity exceeds the capacity of the cathode by 10 ± 2%.

### Battery Assembly and Electrochemical Characterization

### Coin Cells

Two 2016-type coin cells were assembled from each sample foil with metallic lithium as the counter electrode and 1 M LiPF6 (Novolyte Technologies) in 1:1:1 EC/DEC/DMC (ethylene carbonate, Sigma-Aldrich, anhydrous, 99%; diethyl carbonate, Sigma-Aldrich, anhydrous, 99%; dimethyl carbonate, Novolyte Technologies, ≥99%, sealed under nitrogen) as the electrolyte. Cells were first charged at a constant current of 0.1 C until a cutoff voltage of 4.3 V. Consequently, the charging continued with a constant voltage until the current decreased to 0.015 C for the first two cycles. In subsequent charge cycles, the same method was used but the current threshold was raised to 0.02 C. Discharge for the first two cycles was done at a constant current of 0.1 C until 2.6 V was reached, followed by constant voltage discharge until the current decreased to 0.015 C. For a rate test, a subsequent discharge was done to 3.0 V with a different C rate. Cells were tested at 25 °C.

### Pouch Cells

One electrode pair pouch cell (size 44 × 61 mm^2^) was prepared with a graphite anode (Hitachi), an electrolyte of 1.15 M LIPF6 in EC/DMC/EMC (2:4:4), and 1% vinylene carbonate. After the formation cycles, the pouch cells were first charged at a constant current of 1 C until 4.2 V was reached, and after that with a constant voltage until the current decreased to 0.03 C and discharged to 2.5 V at 1 C. Cells were tested at 25 °C. Additionally, every 100 cycles, a capacity check cycle (0.2C/0.2C) was performed. However, for the clarity of the figures, the capacity checks were not plotted. For both coin and pouch cells, the theoretical capacities used to calculate the C rate were 180 and 230 mAh/g for NMC523 and NMC88, respectively. The battery assembly was performed in a dry room at a temperature of 25 °C. The cycling process was conducted with a Maccor Series 4000 battery cycler.

## Results and Discussion

To obtain an understanding of the differences between various materials, solvents, and fabrication methods, the surfaces and cross sections of the fabricated cathodes (before and after calendering) were investigated by scanning electron microscopy (SEM). Figure 2a,b shows a cross-sectional comparison of NMC523 and NMC88 blade-coated with NMP as a solvent and screen-printed with DMF as a solvent on an aluminum current collector. Regardless of the material, solvent, or deposition method, the packing of the NMC spheres is similar. The images demonstrate layer thicknesses of ∼35 to 50 μm and slightly different surface morphologies according to the deposition method. Plane-view SEM images in Figure 2c,d provide more insights regarding the distribution of the material on the surface of the NMC layers. The blade-coated layers demonstrate better confinement of large NMC spheres (Figure 2c), while the screen-printed layers indicate the presence of more unconcealed spherical structures on the surface (Figure 2d). The SEM analysis of the calendered samples (Figure S2a–d) shows that both thickness and roughness of the samples are reduced after calendering, highlighting a more significant reduction for the screen-printed samples.

To quantify the observed topological differences, optical profilometry has been employed. Figure 3 provides the results regarding the surface morphology of the as-printed cathode layers, while Figure S2e–h provides morphology analysis of the electrodes after the calendering process. The combined results of surface roughness for uncalendered and calendered electrodes are presented in Table 2. The blade-coated layers are smoother, with the surface RMS smoothness of 1.885 and 0.613 μm for NMP NMC523 and NMP NMC88, respectively. At the same time, the screen-printed layers demonstrate RMS smoothness of 3.293 and 3.057 μm for DMF NMC523 and DMF NMC88, respectively. Expectedly, calendering reduces the roughness of all electrodes by ∼10 to 30% for blade-coated samples and by 40–50% for screen-printed samples. The information regarding thickness, active material loading, and porosity of the calendered samples is provided in Table S2. Although the thickness of the calendered samples differs, their calculated porosity is very similar, regardless of the material or solvent. The optical profilometry methods have some limitations related to absorptive materials, but the acquired results are in agreement with the SEM findings.

**Table 2:** Surface RMS Roughness (*S*_q_) and Surface Mean Roughness (*S*_a_) of As-Printed and Calendered NMC523 and NMC88 Cathode Layers of a Projected Area of 0.27 mm^2^ Using NMP and DMF Solvents

	NMP NMC523 blade-coated	DMF NMC523 screen-printed	NMP NMC88 blade-coated	DMF NMC88 screen-printed
as-printed *S*_q_ [μm]	1.885	3.293	0.613	3.057
as-printed *S*_a_ [μm]	1.482	2.586	0.462	2.374
calendered *S*_q_ [μm]	1.338	2.039	0.564	1.392
calendered *S*_a_ [μm]	1.010	1.559	0.434	1.063

To better understand the cause of the morphology difference, the DMF-based NMC523 slurry was blade-coated and characterized with SEM and optical profilometry. The results are presented in Figure S1 and Table S1. The blade-coated samples have lower surface roughness regardless of the solvent used. Therefore, we attribute the difference in the surface morphology to particular features of blade coating and screen printing. During blade coating, the doctor blade passes through the surface, enforcing any larger particles into the film. In screen printing, the mesh retracting from the surface of the substrate allows larger slurry particles (NMC spheres) to remain unburied, resulting in a rougher surface after the drying process.

The analysis of solvability suggests that both NMP and DMF fully dissolve the PVDF. However, the NMP-based solution tends to change the color to brown, while the DMF solution remains transparent (Figure S3). This phenomenon is associated with the dehydrofluorination degradation that visually appears as a color change to yellow or amber. These results are in full agreement with the previous research related to solvability of PVDF.^36^ SEM images of the casted PVDF films presented in Figure S3 indicate a very similar behavior of PVDF regardless of the used solvent. An insignificantly higher amount of micropores in the PVDF-DMF film is attributed to an accelerated evaporation of DMF.

**Figure 4:**
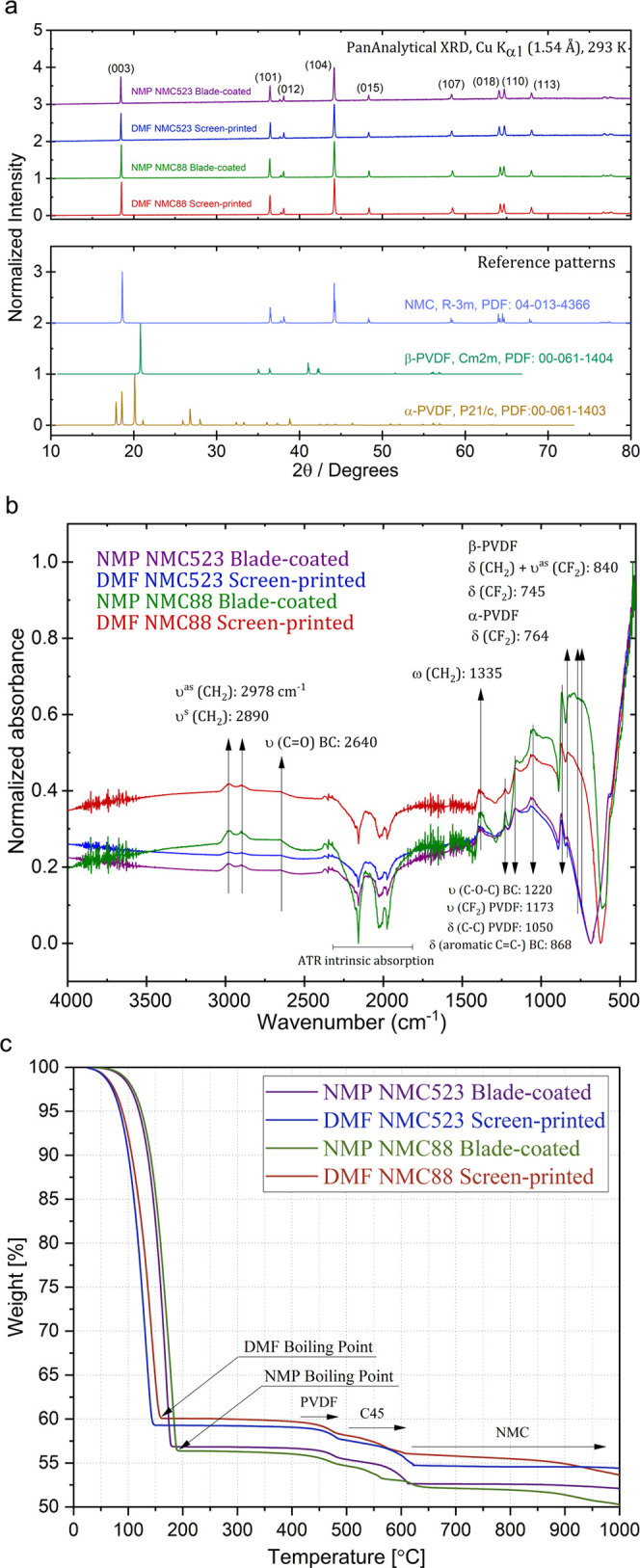
(a) XRD patterns of NMC inks (top) compared with the reference patterns of NMC, α-PVDF, and β-PVDF (bottom). (b) FTIR analysis of NMC materials, PVDF, and CB. (c) TGA analysis of the formulated slurries emphasizes the differences related to different boiling points and densities of the solvents at temperatures up to 200 °C and different compositions of the NMC material at temperatures above 550 °C.

The influence of the solvent replacement and employed printing methods on the fabricated films was further analyzed with XRD and FTIR. The XRD patterns of all inks (Figure 4a) show the expected (hkl) reflections of a rhombohedral NMC (LiMnCoNiO_2_), thus confirming the expected crystalline structure of the cathode material.^43^ Expectedly, we have not noticed any peak related to the reflections of PVDF, nor any significant broadening of the peaks due to the amorphous structure of carbon black. Such results are expected since the concentration of NMC is much higher than that of PVDF and carbon black. Therefore, the consequent higher intensity of the NMC peaks suppresses the XRD peaks of the other materials. Although it is possible to notice a slight increment in intensity in the background toward higher degrees in 2Θ, the Rietveld (RIR) refinement applied in the diffractograms indicated 100% of NCM in all of the samples.

The FTIR spectra of all samples (Figure 4b) show similar bands related to the vibrational modes of carbon black (CB), α-PVDF, and β-PVDF. The D_3d_^5^ spectroscopic symmetry of NCM gives rise to seven vibrational modes (4A_2u_ + 3E_u_); however, the corresponding bands are seen in the spectral range between 670 and 235 cm^–1^, which is not well resolved or out of the detection range of the utilized equipment.^44^ The bands centered at 2978 and 2890 cm^–1^ are related to the asymmetric and symmetric stretching of the −CH_2_ groups of PVDF being observed in a similar position for all samples.^45^ The weak bands with a higher intensity at 2640 cm^–1^ are related to the stretching of the −C=O bonds of CB.^46^ The sharp bands noticed at 1335 cm^–1^ are assigned to the bending vibrations out-of-plane on the −CH_2_ groups of PVDF.^47^ The following bands at 1220, 1173, 1050, and 868 cm^–1^ are related to the stretching of −COC groups of CB, symmetrical stretching of the −CF_2_ groups of PVDF, bending of the–C–C groups of PVDF, and bending of the conjugated −C=C groups of BC, respectively.^46,48,49^ All of the IR bands detailed above were observed in the same spectral range for all samples. The following bands show small differences between the samples NMC88 and NMC523, which may indicate changes in the isomeric conformations of the PVDF according to concentration changes in these systems. The band characteristics of β-PVDF are seen at 840 cm^–1^ (bending of −CH_2_ coupled with asymmetrical stretching of −CF_2_) and 745 cm^–1^ (bending of −CF_2_), appearing more prominent in the NMC523 samples, while the broad bands at 764 cm^–1^ are assigned to the bending of −CF_2_ groups of α-PVDF being better observed in the samples of NMC88.^47,49,50^

The formulated slurries were characterized by thermogravimetric analysis (TGA) that provided information regarding the influence of the material and solvent on the slurry behavior. Figure 4c depicts the change in slurry weight as a function of temperature. Expectedly, the DMF-based slurries lose their weight faster than NMP-based slurries due to the different boiling points of these two solvents (NMP 203 °C, DMF 153 °C). Another difference is related to the variation of the level of plateau after solvent evaporation. For DMF-based slurries, the plateau settles at ∼60% of the total weight, while for NMP-based slurries, the plateau establishes at ∼57% of the total weight. Expectedly, the divergence comes from different densities of the used solvents (NMP, 1.03 g/cm^3^; DMF, 0.944 g/cm^3^). Although it is not critical from the perspective of slurry drying at normal battery-fabricating conditions, the plots indicate material-related differences at temperatures above 550 °C. This difference is associated with different NMC ratios (50:20:30 vs. 88:03:09) and different boiling points of the elements that compose NMC (Ni—1453 °C, Mn—1244 °C, Co—1495 °C). Higher amount of high boiling point Ni and lower amount of lower boiling point Mn in NMC88 stabilize its weight for a longer time after the initial drop at ∼550 °C.

**Figure 5:**
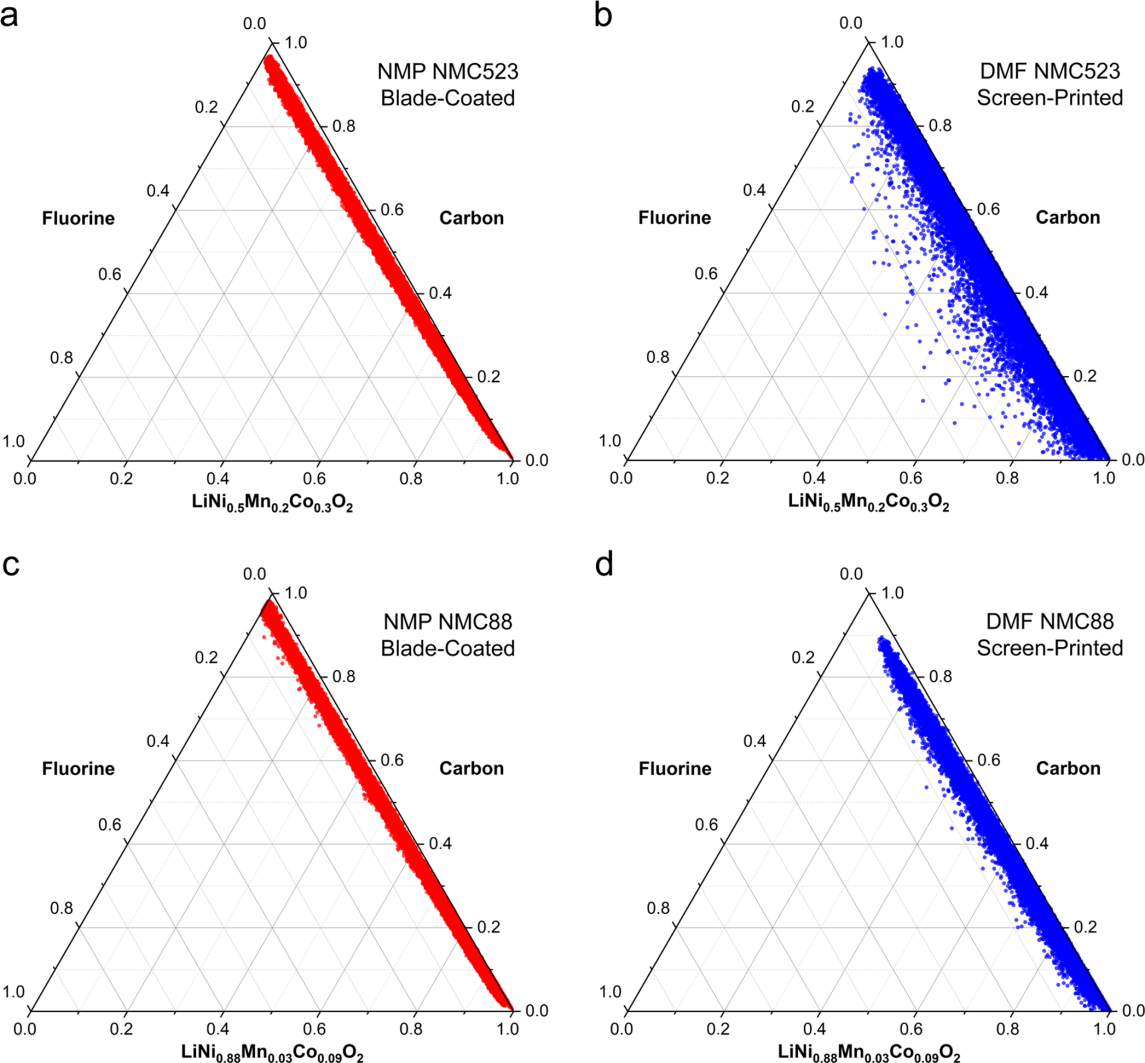
Ternary plots representing the distribution of C, F, and NMC in the as-printed samples performed with an electron probe microanalyzer. (a) Elemental distribution for NMP NMC523 blade-coated—the overwhelming amount of NMC is accompanied with evenly distributed C and F. (b) Elemental distribution for DMF NMC523 screen-printed—the plot indicates high amount of NMC complemented with uneven distribution of F, while C remains rather stably distributed. (c) Elemental distribution for NMP NMC88 blade-coated—as in panel (a), an overwhelming amount of NMC is accompanied by a fairly even distribution of F and C. (d) Elemental distribution for DMF NMC88 screen-printed—the plot indicates a high amount of NMC complemented with rather even distribution of F and insignificant amount of areas with C deficiency.

To compare the differences in elemental distribution between various samples, the electron probe microanalyzer (EPMA) in wavelength dispersive spectrometry (WDS) mode was used. The most important advantage of this method is that it can conduct reliable characterization of rough samples and has much better energy resolution and high reproducibility. In contrast, the commonly used SEM-EDX method suffers from low energy resolution, poor reproducibility, and difficulties in appropriate quantitative precision in rough samples. The generic elemental mapping is provided in Figures S4–S7. However, to provide more quantitative distribution and to better recognize differences, we transposed the mapping data into ternary plots with emphasis on carbon, fluorine, and combined NMC distribution (Figure 5). In these plots, the ratios of the three variables (C, F, and NMC) are depicted as positions in an equilateral triangle. Expectedly, the plots indicate very high ratios of the NMC material in the composition, while C and F remain at low ratios. Interestingly, the DMF screen-printed samples show that fluorine distribution differs from the blade-coated samples. It is especially distinguishable for the NMC523 material where there are some locations with over 20% of fluorine content. Also, as opposed to the blade-coated samples, the screen-printed ones contain areas without carbon. One of the aspects contributing to such behaviors could be the higher surface roughness of the screen-printed layers due to the relatively coarse stencil mesh. Another aspect involves lower surface tension of DMF that promotes binding of carbon black to the PVDF binder instead of the surface of the NMC.^42^ The lower surface tension of DMF might accelerate sedimentation of the NMC, affecting the distribution of the materials. The brown color of the NMP-based PVDF solution indicates the gelation of the PVDF solution (due to the formation of cross-links between adjacent PVDF chains) that provides protection against sedimentation of the NMC particles and allows more even distribution of the materials.^36,51^

**Figure 6:**
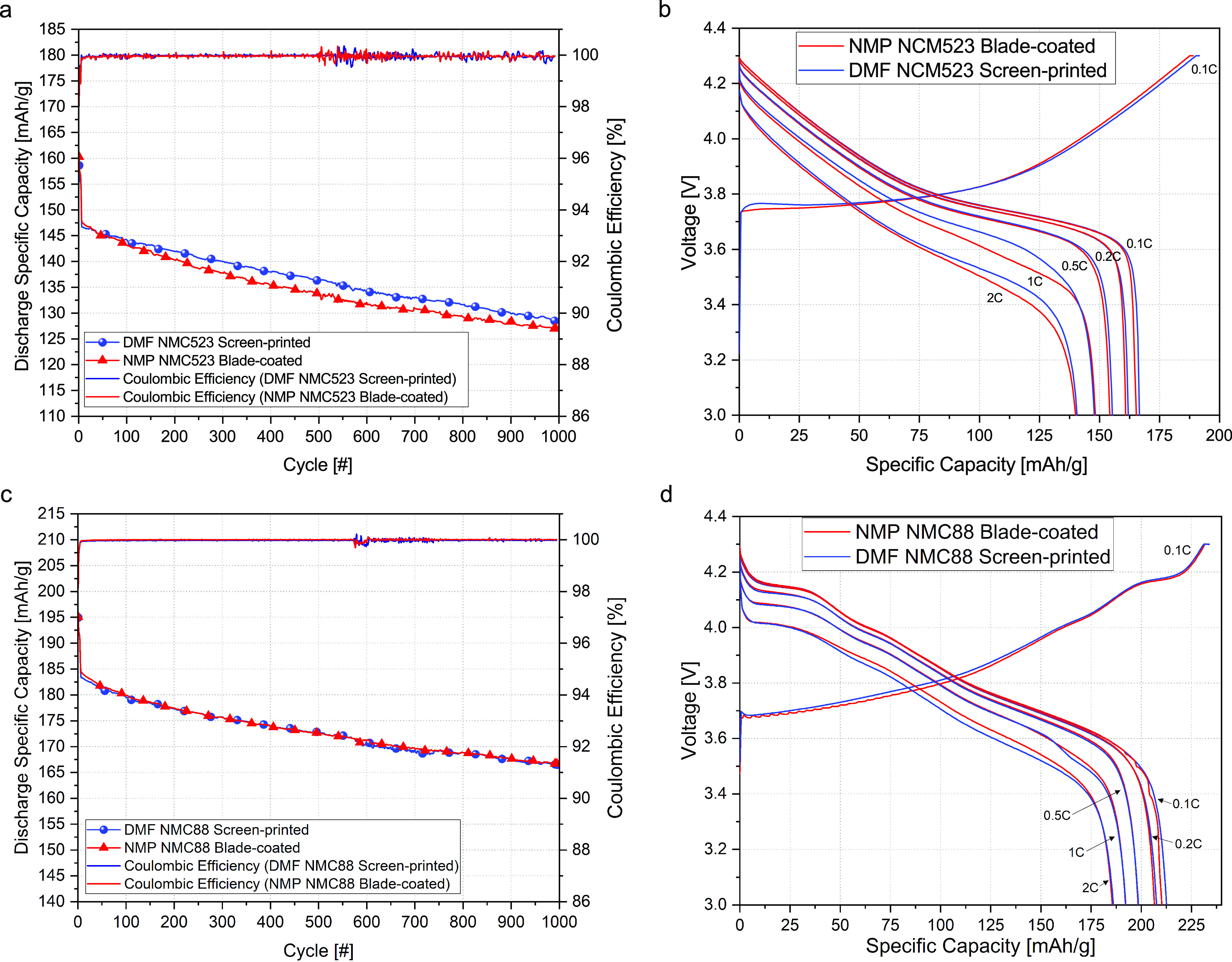
Electrochemical characterization of fabricated batteries. (a) Influence of cycling on retaining the capacity of pouch batteries using DMF NMC523 screen-printed and NMP NMC523 blade-coated cathodes. Coulombic efficiency of the pouch batteries with their respective cathodes. (b) Comparison of the impact of the fabrication method and solvent on the specific capacity of NMC523 coin-cell batteries at various discharge rates. (c) Capacity retention and Coulombic efficiency of batteries with differently fabricated NMC88 cathodes. (d) Impact of the fabrication method and solvent on specific capacity of NMC88 coin-cell batteries at various discharge rates.

The blade-coated and screen-printed cathodes, after calendering, were used to fabricate coin and pouch batteries that underwent electrochemical characterization and cycling process. Figure 6a depicts the influence of cycling on the discharge specific capacity of pouch batteries using an NMC523 cathode material. In addition, the Coulombic efficiency is plotted. The plots indicate a decrease of specific capacity by ∼13% after 1000 charge/discharge (1C/1C) cycles, from 147 to 128 mAh/g. In addition, the plots suggest that the performance of the DMF NMC523 screen-printed battery is similar to its NMP NMC523 blade-coated cells. For both NMP NMC523 blade-coated and DMF NMC523 screen-printed, the Coulombic efficiency oscillates around 100%. Importantly, the increased variation (±0.5%) between 500th and 700th cycles was caused by a laboratory air conditioning system malfunction. Figure 6b shows the behavior of the cell batteries during charging and at various discharging rates in the range of 0.1C–2C. Although the charging plots are almost the same for the tested NMC523 samples, the discharge curves indicate slightly better performance of DMF NMC523 screen-printed batteries at higher discharge currents (1 C and 2 C), which is attributed to lower thickness of the DMF screen-printed cathodes. A similar set of electrochemical analysis was conducted for the NMC88 material. The cycling tests, depicted in Figure 6c, indicate a similar behavior of the NMC88 batteries—decrease of the specific capacity by 10%, from 183 to 166 mAh/g after 1000 cycles, regardless of the fabrication method and used solvent. The Coulombic efficiency remains stable at almost 100%, with insignificant oscillation caused by temperature variation between 570th and 770th cycles. Figure 6d shows a superior capacity of the batteries with the NMC88 cathode material. At low discharge rates (0.1 C), the capacity reaches 213 mAh/g. Importantly, the specific capacity of the batteries is not affected by the fabrication method or solvent.

To estimate the difference in drying time for NMP- and DMF-based NMC cathodes, we calculate the drying ratio based on a model with the following assumptions: slurry of NMC + C45 + PVDF (ratio 92:4:4), solvent concentration of 1.27 g/mL, deposited rectangle of 20 × 10 cm^2^, and thickness of 100 μm. In terms of volume, the solvent represented ∼77% of the slurry volume. The evaporation rate of the solvent, *R*_evap_, was calculated using eq 1(52)
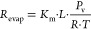
1where *K*_m_ is the mass transfer coefficient, *L* is the length of the drying film, *P*_v_ is the vapor pressure of the solvent, *R* is the universal gas constant, and *T* is the absolute temperature. Further details of the calculations are provided in the Supporting Information. Our model indicates that a 100 μm thick rectangle of NMP-based NMC slurry requires 671 s of drying at a temperature of 100 °C, while the same rectangle with DMF-based NMC slurry needs only 143 s at 100 °C. This calculation shows an over fourfold reduction in drying time, favoring DMF over NMP. Considering the data reported by Davidsson that fabrication of a 100 kWh battery (without raw material processing) consumes 5–6.5 MWh of energy and solvent drying/recovery stands for ∼40% of the total energy (for NMP), the drying/recovery consumes 2–2.6 MWh.^8,10^ Replacement of NMP with DMF offers fourfold reduction of energy needed for solvent drying/recovery. Therefore, the total energy needed to fabricate a 100 kWh pack will be reduced to 3.5–4.55 MWh, representing ∼30% reduction of energy demand. Although the provided values are just an approximation, they are in line with similar studies that estimated 43% reduction of energy consumption using water as a solvent.^53^

The results reveal that the proposed method of using screen printing and DMF allows the fabrication of batteries without compromising their performance. Importantly, the low boiling point and high autoignition temperature of DMF enable energy-efficient and safe fabrication of NMC cathodes.

Compared to the NMP blade-coated samples, the DMF screen-printed ones demonstrate increased roughness and differences in the material distribution. At the same time, these factors do not significantly affect the electrochemical performance of the fabricated batteries. An important factor that needs to be taken into account is the calendering that reduces the roughness and improves the long-range electrical contacts.^54^

The FTIR results suggest a need for further investigation of occurrence of β-PVDF, which is more prominent in the NMC523 samples and might be one of the contributors affecting the battery performance. In addition, future works shall focus on the analysis of different green solvents, their printing compatibility, and more extensive cycling and higher charge/discharge rates that might be especially attractive for the EV industry. Naturally, screen-printing compatibility of the proposed method enables another avenue of battery development related to the implementation of cathodes of various application-designed shapes.

## Conclusions

This research demonstrates that the proposed method of screen printing associated with the usage of DMF as a replacement for blade coating and NMP is a valuable alternative that provides similar results, and it benefits from the advantages of using DMF and screen printing. Both the NMP blade-coated and DMF screen-printed batteries show superior performance, retaining 87 and 90% of their capacity after 1000 (1C/1C) cycles. The physicochemical analysis reveals that the DMF-based, screen-printed cathodes possess increased roughness and slightly uneven distribution of the PVDF, factors which, however, do not negatively affect the battery performance due to the calendering process prior to battery assembling. The usage of NMC523 and NMC88 emphasizes the universality and high applicability of the proposed method. Although DMF does not belong to a group of “green” solvents and further research is needed, this study demonstrates that usage of suitable low-boiling-point solvents will benefit the battery industry by reducing the cell manufacturing energy consumption by ∼30%, supporting the reduction of greenhouse gas emission during battery fabrication.

## Supporting Information Available

Additional morphology analysis for explaining the causes of the identified differences; morphology visualization of calendered samples for various solvents and NMC materials; parameters of calendered cathodes for various solvents and active materials; morphology of PVDF thin films diluted in NMP and DMF, casted on an aluminum foil; details of the calculations used to estimate the energy consumption during the drying process for NMP- and DMF-based slurries; images of the elemental distribution, acquired with a field emission electron probe microanalyzer; figures comparing the average data from the battery cycling tests for NMP NMC523 blade-coated, NMP NMC88 blade-coated, DMF NMC523 screen-printed, and DMF NMC88 screen-printed; and morphological analysis of cyrene-based printed NMC electrodes (PDF)
