# Supplementary material for: Suitable Cathode NMP Replacement for Efficient Sustainable Printed Li-Ion Batteries
Source: ACS Appl Energy Mater. 2022 Mar 29;5(4):4047–58. doi: 10.1021/acsaem.1c02923 (PMC9045678; doi:10.1021/acsaem.1c02923)
Supplement: Supplementary file 1 — ae1c02923_si_001.pdf [file ae1c02923_si_001.pdf]

Supporting Information

Suitable cathode NMP replacement for efficient sustainable printed Li-ion batteries

Rafal Sliz *†, Juho Valikangas ‡, Hellen Silva Santos §, Pauliina Vilmi †, Lassi Rieppo ⸙, Tao Hu ‡, Ulla Lassi ‡, Tapio Fabritius †

† Optoelectronics and Measurement Techniques Unit, University of Oulu, 90570 Oulu, Finland

‡ Research Unit of Sustainable Chemistry, University of Oulu, 90570 Oulu, Finland

§ Fibre and Particle Engineering Research Unit, University of Oulu, 90570 Oulu, Finland

⸙ Research Unit of Medical Imaging, Physics and Technology, University of Oulu, 90570 Oulu, Finland

Corresponding author: Rafal Sliz, rafal.sliz@oulu.fi

1. Additional morphology analysis

To verify the causation of the different morphology, additional DMF-based slurries of NMP523 were blade-coated, and their properties were analyzed (Figure S1).

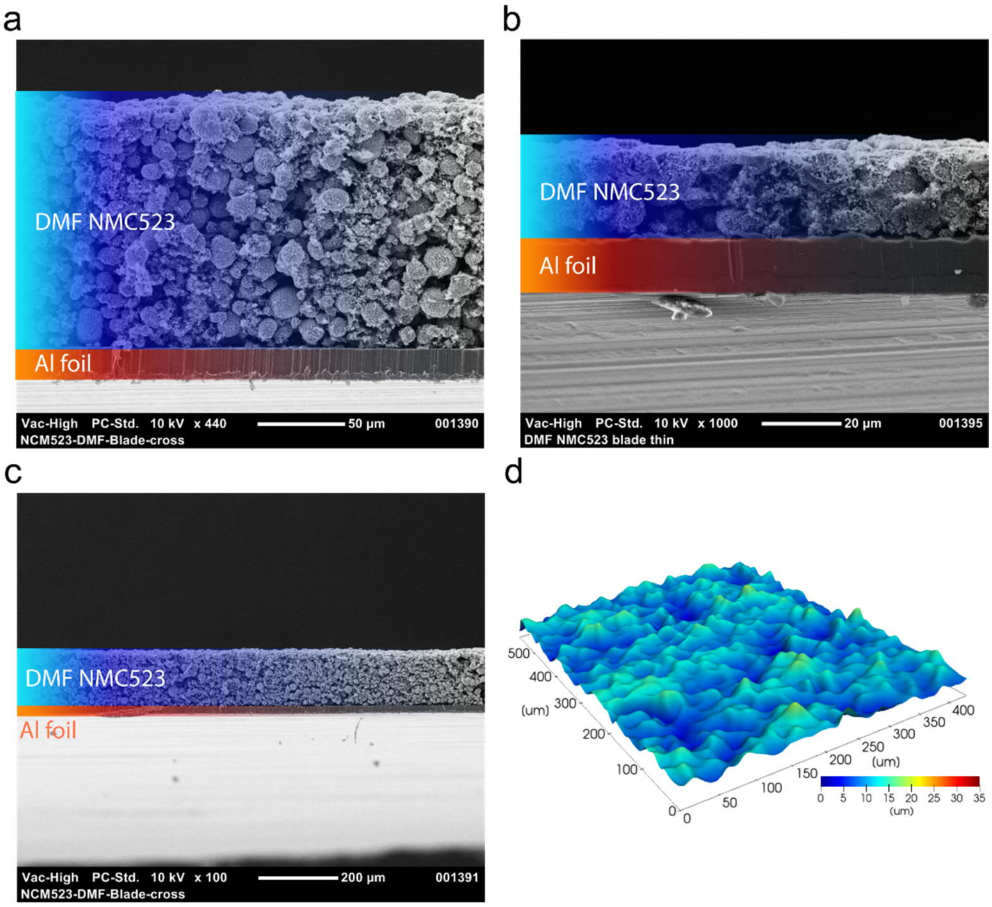

Figure S1. Roughness analysis of DMF blade-coated samples. a) Cross-sectional SEM image of a thick DMF NMC523 layer on Al current collector. b) Cross-sectional SEM image of a thin DMF NMC 523 layer on Al current collector. c) A lower SEM magnification image depicting the smoothness of the Blade-coated DMF NMC523.

Table S1. Additional comparison of surface RMS roughness (S_q) and surface mean roughness (S_a) of DMF NMC523 Blade-coated layer with the remaining samples.

	DMF NMC523 Blade-coated	NMP NMC523 Blade-coated	DMF NMC523 Screen-printed	NMP NMC88 Blade-coated	DMF NMC88 Screen-printed
Sq [µm]	2.189	1.885	3.293	0.613	3.057
Sa [µm]	1.732	1.482	2.586	0.462	2.374

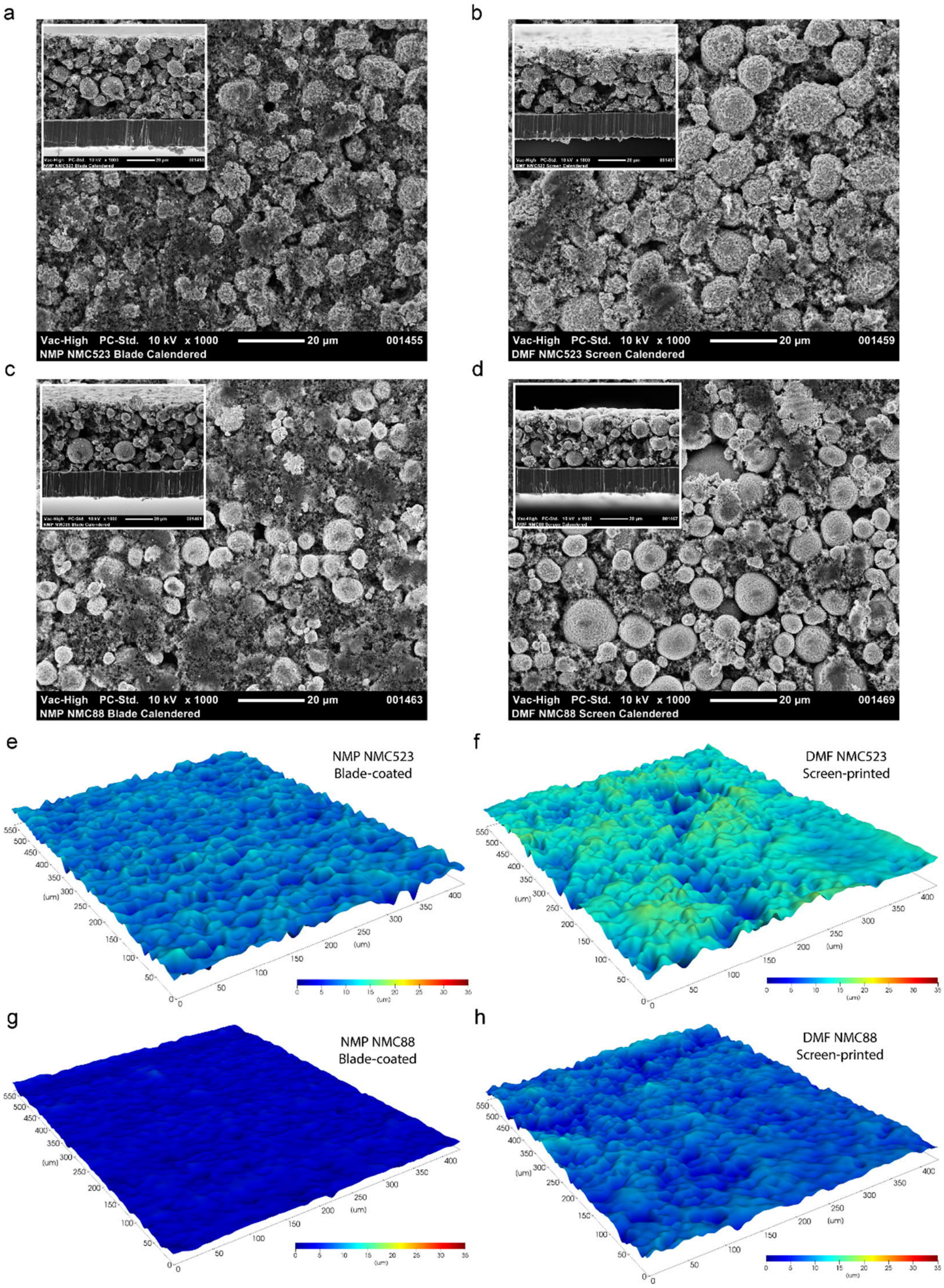

Figure S2. a-d) SEM analysis of the calendered electrodes for various solvents and NMC materials. Insets demonstrate cross-sections of the electrodes on the Al electrodes. e-h) Surface morphology of calendered samples.

Table S2. Calendered cathode parameters for various solvents and active materials.

Cathode	Active material loading [mg/cm^2]	Cathode Thickness [μm]	Porosity [%]
NMP NMC523 Blade-Coated	14.0	57	42.4
DMF NMC523 Screen-Printed	8.6	36	44.1
NMP NMC88 Blade-Coated	11.1	45	42.4
DMF NMC88 Screen-Printed	8.7	36	43.3

Electrode porosity calculation

The porosity of the NMC electrode without aluminum substrate was calculated as follows:

$$Theoretical\ density = \frac{1}{\left(\frac{Material\ wt.\%}{Material\ density}\right)} \quad (S1)$$

$$Actual\ density = \frac{Electrode\ mass\ loading}{Electrode\ Thickness} \quad (S2)$$

$$Porosity = \left(1 - \frac{Actual\ density}{Theoretical\ density}\right) \cdot 100\,\% \quad (S3)$$

The following theoretical material densities were used:

- NMC: 4.85 g/cm^3
- PVDF: 1.8 g/cm^3
- Carbon Black: 0.16 g/cm^3

Although there is a difference in the electrode loading that might affect the performance of the batteries, the difference is more prominent for high C-rates.[1] Hu et al. investigate the influence of electrode thickness and porosity on the battery's performance.[2] In this report, Hu et al. show that at discharge rates of 0.1C to 2C, the cathode loadings of 9.55 mg/cm^2 and 14.15 mg/cm^2 demonstrate almost identical cycling performance. In addition, this publication emphasizes the importance of the cathode porosity on the performance of the battery. In our experiments the porosity of the cathodes was 43.05 % with standard deviation of 0.81 %. This value (~43 %) allows full penetration of the ionic liquid into the pores of the cathode, improving the ion transfer and reducing the influence of the cathode loading/thickness. The performance is affected by the thickness (active material loading) of the electrodes, however, our experiments remain in the regime where this influence is relatively small (cathode loading below ≤14.00 mg/cm^2 and discharge rates of ≤2C).

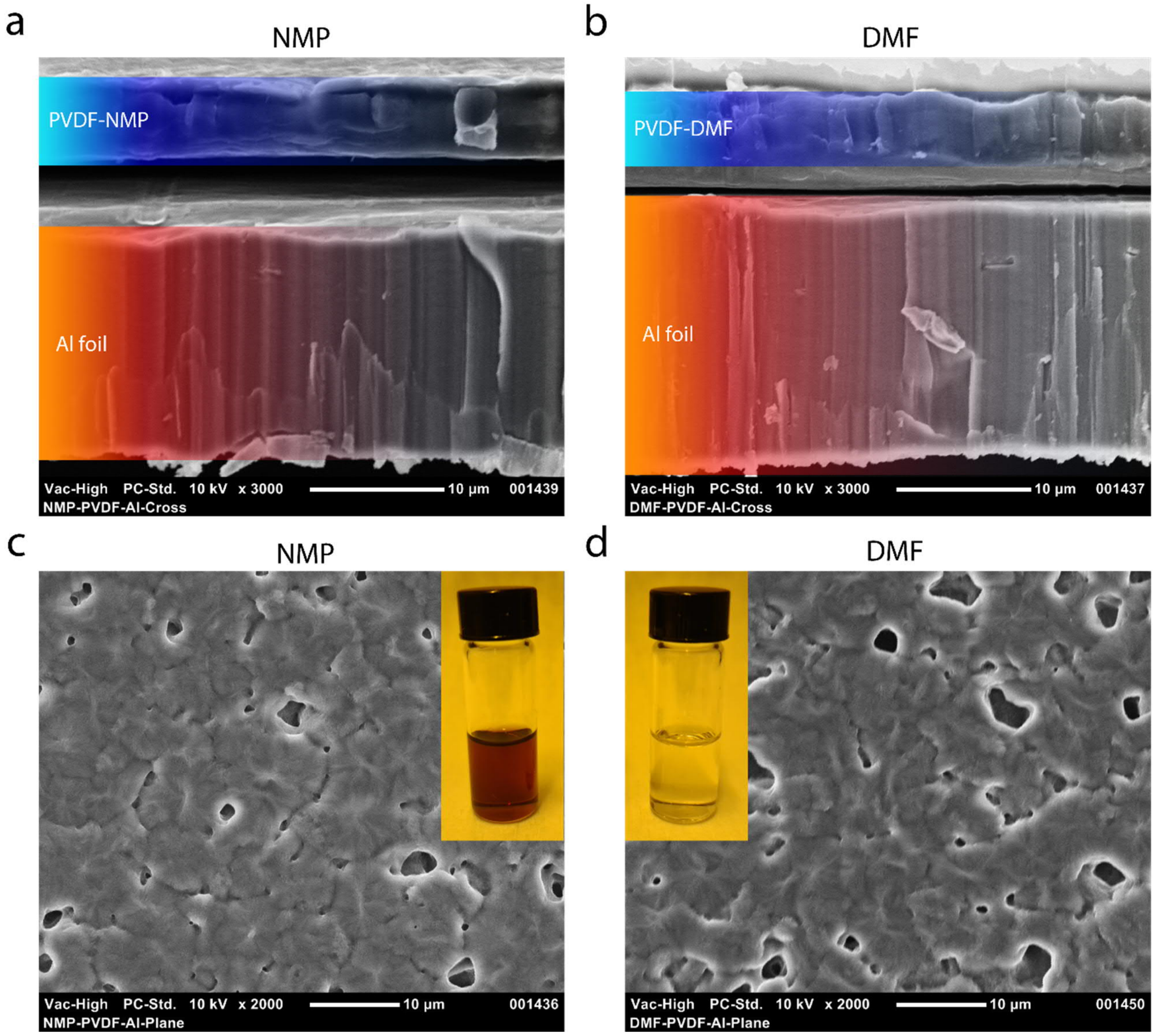

Figure S3. Morphology of PVDF thin-films diluted in NMP (a and c) and DMF (b and d), and casted on aluminum foil. Insets demonstrate the behavior of the PVDF in the solvents.

2. Drying energy consumption calculations

To estimate the difference in energy consumption for NMP- and DMF-based NMC cathodes, we calculated the drying ratio based on the following model. A slurry of NMC+C45+PVDF (ratio 92:4:4) was mixed with the solvent at concentrations 1.32 g/ml and 1.22 g/ml for DMF and NMP, respectively. Consequently, the slurry was deposited as a rectangle of 20 x 10 cm and thickness 100 µm. In terms of volume, the solvent was representing approximately 77 % of the slurry volume. The evaporation rate of the solvent, R_{evap}, was calculated using equation (S4).[3]

$$R_{evap} = K_m \cdot L \cdot \frac{P_v}{R \cdot T} \quad (S4)$$

Where K_m is the mass transfer coefficient, L is the length of the drying film, P_v is the vapor pressure of the solvent, R is the universal gas constant, and T is the absolute temperature. The following vapor pressure values at 100 ºC were used: 3.45 kPa and 21.85 kPa for NMP and DMF, respectively. To determine the value of K_m, we calculated the Sherwood number Sh (Formula S5), using Reynolds and Schmidt numbers.

$$Sh = \frac{K_m \cdot L}{D} = 0.664 \cdot \sqrt{Re} \cdot \sqrt[3]{Sc} \quad (S5)$$

Where D is the diffusion coefficient of solvent in air, Re is the Reynolds number, and Sc is the Schmidt number calculated according to the equations S6 and S7, respectively. The diffusion coefficients for the solvents were calculated using an equation derived by Hirschfelder et al. based on Chapman–Enskog theory.[4–6] The following values of diffusion coefficient were used in calculating the Sherwood number: $1.25 \cdot 10^{-1}$ and $1.12 \cdot 10^{-1}$ cm^2/s for NMP and DMF, respectively.

$$Sc = \frac{\eta}{D} \quad (S6)$$

Where η is the kinematic viscosity of the air: 0.229 cm^2/s at 100 ºC.

$$Re = \frac{U \cdot L}{\eta} \quad (S7)$$

Where U is the air velocity, assumed as constant (0.05 m/s) for both solvents.

The evaporation rate for NMP and DMF were $2.36 \cdot 10^{-5}$ and $1.39 \cdot 10^{-4}$, respectively. Accordingly, the drying times considering the molecular weights of the solvents were calculated.

3. Mapping of cathode surface with Field Emission Electron Probe Microanalyzer

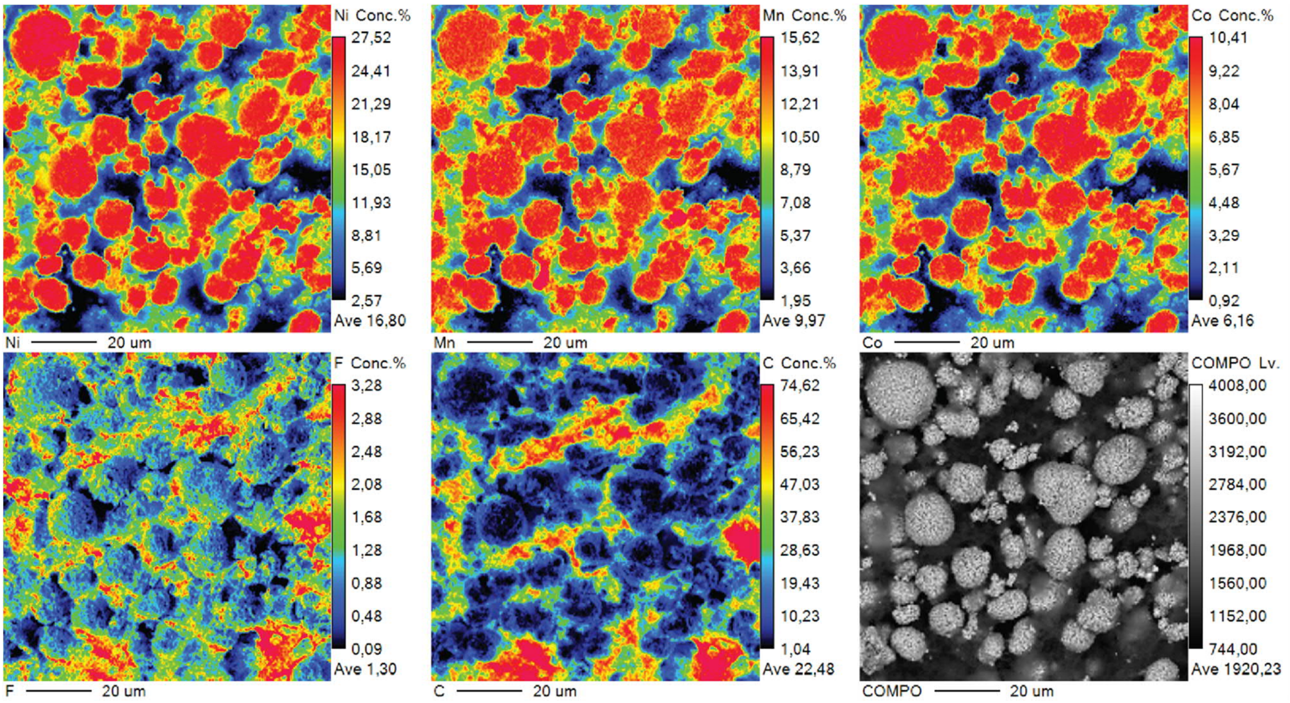

Figure S4. Material distribution on the surface of the NMP NMC523 Blade-coated analyzed with Field Emission Electron Probe Microanalyzer.

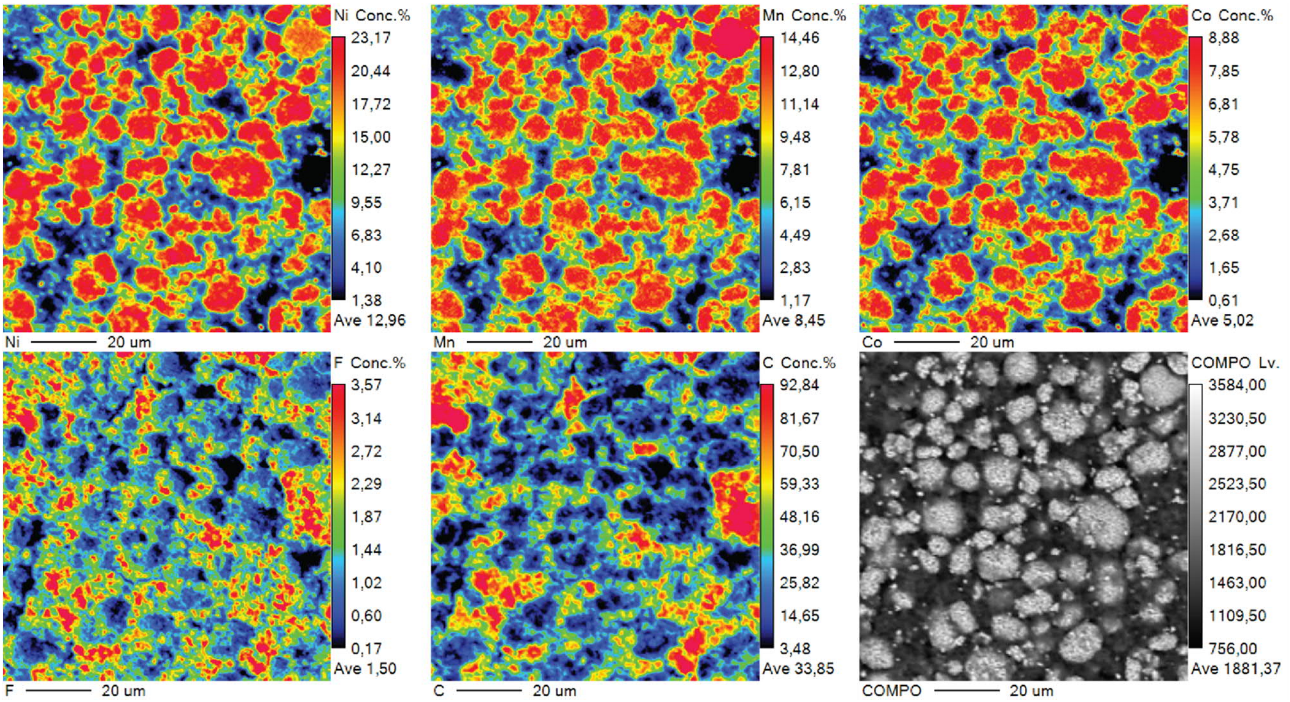

Figure S5. Material distribution on the surface of the DMF NMC523 Screen-printed analyzed with Field Emission Electron Probe Microanalyzer.

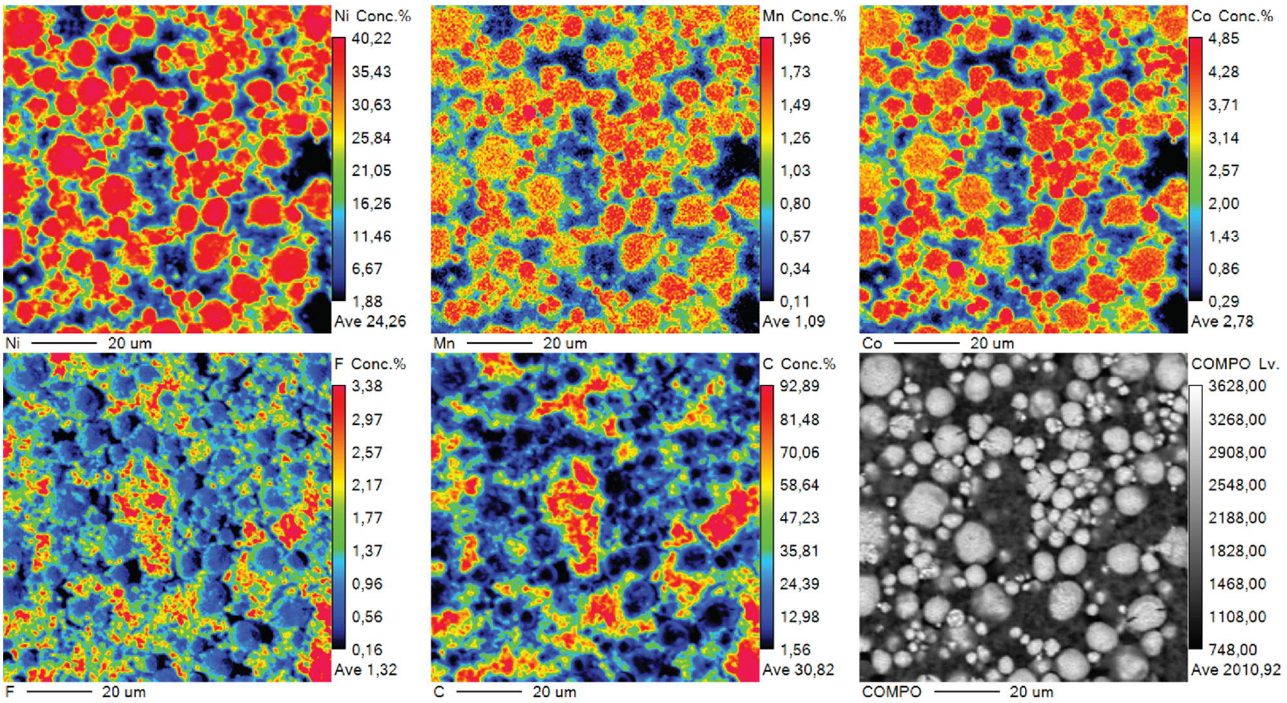

Figure S6. Material distribution on the surface of the NMP NMC88 Blade-coated analyzed with Field Emission Electron Probe Microanalyzer.

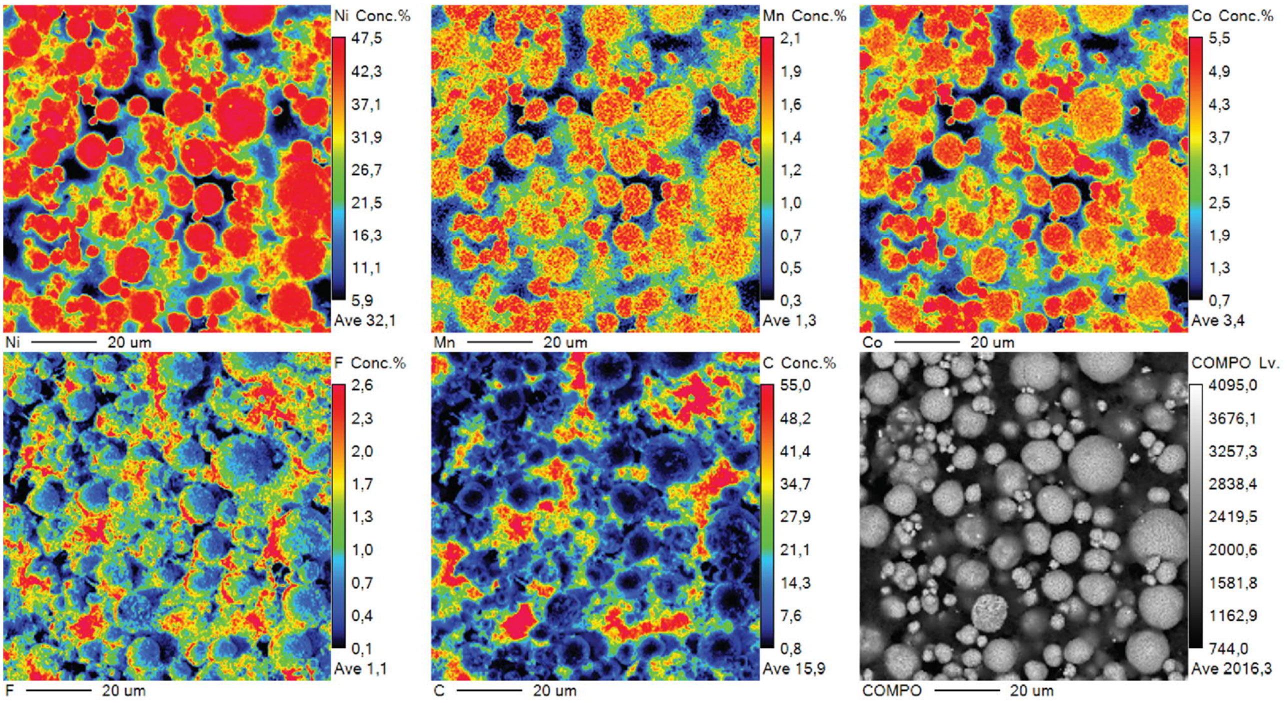

Figure S7. Material distribution on the surface of the DMF NMC88 Screen-printed analyzed with Field Emission Electron Probe Microanalyzer.

4. Additional pouch battery cycling results

Comparison of cycling of two DMF screen-printed cells with the best NMP blade-coated cells for NCM523 and NMC88 is depicted in Figure S8. For each NMC material, the discharge specific capacities of two DMF screen-printed pouch batteries were averaged, and standard deviation calculated. The average value (blue spheres) and the standard deviation (green area) are plotted and compared with the best NMP blade-coated poach batteries (red squares).

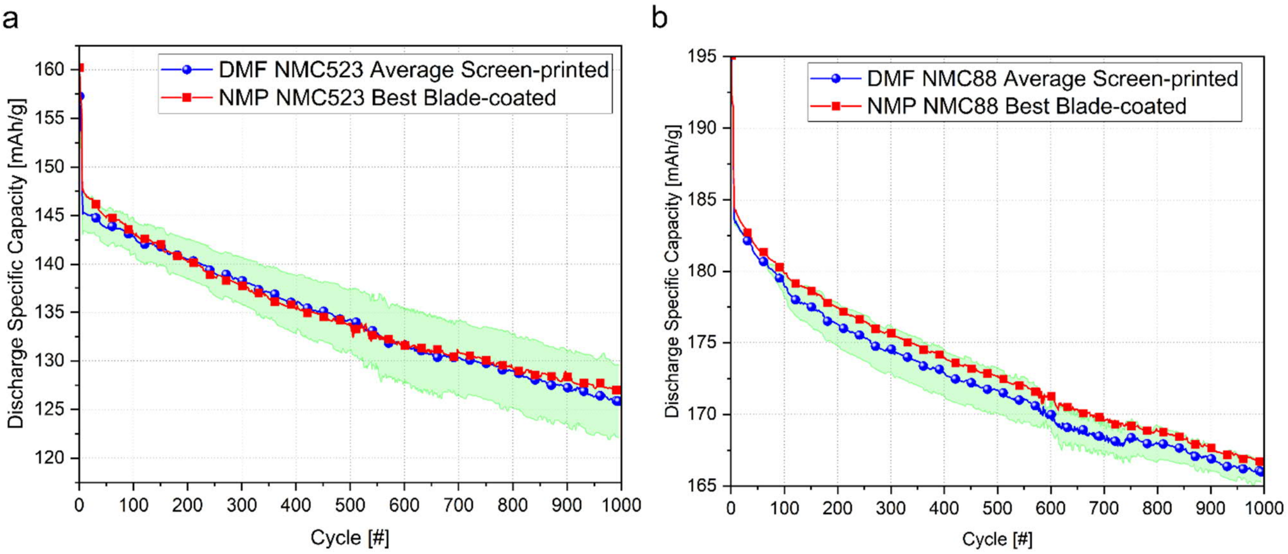

Figure S8. Comparison of charge/discharge cycling of the best NMP blade-coated pouch batteries with their screen-printed averaged counterparts. The green area in the plots represents the standard deviation. a) Comparison for NMC523 cathode material. b) Comparison for NMC88 cathode material.

5. Cyrene based cathode – delamination from the aluminum current collector

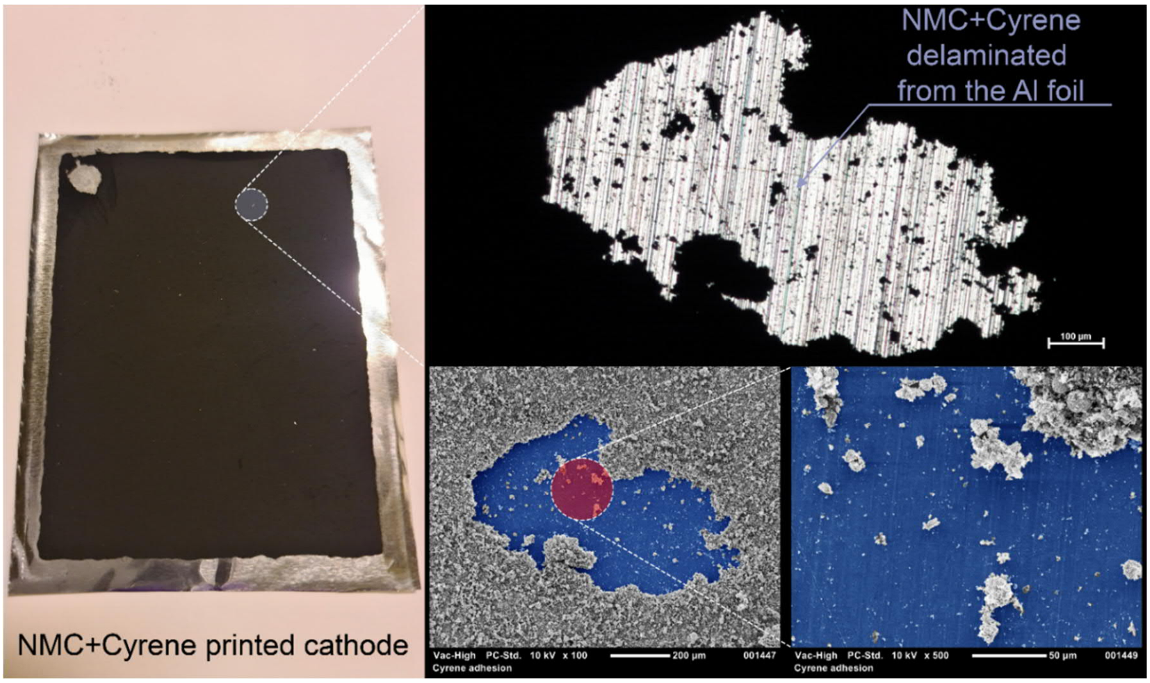

Figure S9. Exemplary screen-printed NMC88 cathode that utilized Cyrene as solvent. After drying, the NMC material spontaneously delaminates from the aluminum collector. Bending or gentle touch cause significant delamination (the upper left-hand corner).

6. References

(1) Xu M and Wang X 2017 Electrode Thickness Correlated Parameters Estimation for a Li-Ion NMC Battery Electrochemical Model *ECS Trans.* ***2017***, 77, 491–507, https://doi.org/10.1149/07711.0491ECST/XML

(2) Hu J, Wu B, Cao X, Bi Y, Chae S, Niu C, Xiao B, Tao J, Zhang J and Xiao J 2020 Evolution of the rate-limiting step: From thin film to thick Ni-rich cathodes *J. Power Sources,* **2020**, 454 227966, https://doi.org/10.1016/J.JPOWSOUR.2020.227966

(3) Shargaieva, O.; Näsström, H.; Smith, J. A.; Többens, D.; Munir, R.; Unger, E. Hybrid Perovskite Crystallization from Binary Solvent Mixtures: Interplay of Evaporation Rate and Binding Strength of Solvents. *Materials Advances* **2020**, *1*(9), 3314–3321. https://doi.org/10.1039/D0MA00815J.

(4) Fundamentals of Momentum, Heat and Mass Transfer, 6th Edition, January **2014** | Wiley

(5) Bird, R. B. Theory of Diffusion. *Advances in Chemical Engineering* **1956**, *1* (C), 155–239. https://doi.org/10.1016/S0065-2377(08)60312-9.

(6) Hirschfelder, J. O.; Bird, R. Byron.; Spotz, E. L. The Transport Properties of Gases and Gaseous Mixtures. II. *Chemical Reviews* **2002**, *44* (1), 205–231. https://doi.org/10.1021/CR60137A012.
